# Siamese meta-learning network for social disputes based on multi-head attention

**DOI:** 10.7717/peerj-cs.2910

**Published:** 2025-06-04

**Authors:** Jing Wang, Rui Zhang, Huijian Han, Yuxiang Liu, Zhaoxing Peng

**Affiliations:** School of Computer Science and Technology, Shandong University of Finance and Economics, Jinan, China

**Keywords:** Few-shot text classification, Siamese network, Multi-head attention, Meta-learning, Synonym replacement

## Abstract

Few-shot learning has been widely used in scenarios where labeled data is scarce, where meta-learning based few-shot classification is widely used, such as the Siamese network. Although the Siamese network has achieved good results in some applications, there are still some problems: (1) When computing prototype vectors with external knowledge of class labels, it depends on the quality and correctness of class labels. (2) When processing data, the Siamese network is not sufficient to capture dependencies between long distance. (3) When the data is complex or the samples are unbalanced, the Siamese network does not achieve the best performance. Therefore, this article proposes a multi-head attention siamese meta-learning network (MASM). Specifically, this article uses synonym substitution to solve the problem that the computation of prototype vectors will be transitionally dependent on class label. In addition, we use the multi-head attention mechanism to capture long-distance dependence by exploiting its global perception capability, which further improves the model performance. We conducted experiments on four benchmark datasets, all of which achieved good performance, and also applied the model for the first time in the field of social disputes, and experimented on a homemade private dispute dataset, which also achieved good results.

## Introduction

Text classification technology, as one of a wide range of analysis tools, has achieved remarkable results in the fields of news classification (Blackman, 2013), sentiment analysis (Liu, 2010), and information retrieval (Mitra & Craswell, 2017). However, in the field of social disputes, some data involves sensitive contents such as personal rights and interests and organizational conflicts. For reasons of privacy protection, commercial secrets, and reputation concerns, some dispute-related information is strictly confidential, or some parties are unwilling to disclose it, thus resulting in extremely limited publicly accessible data. The conflict and dispute data are usually irregular because this kind of data usually includes all kinds of sources, such as records of verbal conversations, complaint letters, and Internet forum discussions. Data from different sources have significant differences in language styles, expressions, information details, *etc*., which greatly increases the complexity of data preprocessing and puts higher requirements on the applicability and generalization ability of the classification model. Therefore, the traditional model of text classification based on large amounts of data is facing significant challenges in the field of social disputes.

Therefore, few-shot text classification shows unique advantages and applicability in social disputes. In previous studies, meta-learning was the main method of few-shot learning. Representative meta-learning methods are prototypical networks (Snell, Swersky & Zemel, 2017) and Siamese network (Liu et al., 2022). In this article, we use the meta learning Siamese network as the base model, because the model no longer estimates the prototype samples from the sampled support set, but utilizes the external knowledge to compute it, which eliminates the dependence on the randomly sampled support set. And in this model, the samples and prototype vectors are mapped into a low-dimensional space, where the distance between the prototype vectors between different classes is increased, while the distance between the samples and the corresponding prototype vectors is shortened to improve the classification performance.

Although the meta Learning Siamese network model has been successful in the field of few-shot classification, there are still some problems: (1) In the meta learning Siamese network model, the prototype vectors are computed using external knowledge on the class labels, but they are dependent on the quality and accuracy of the class labels. (2) Meta learning Siamese network may not achieve the optimal performance when the dataset is complex or the samples are unbalanced. (3) When dealing with textual data, traditional Siamese networks may not be sufficient to capture dependencies between long distance.

To solve the above problems, this article proposes multi-head attention Siamese meta-learning (MASM). In this article, when constructing prototype vectors, we use the information strategy of synonym substitution to improve the deficiency of relying only on external knowledge of class labels when computing prototype vectors. First, we obtain a dictionary of synonyms from WordNet (Fellbaum & Miller, 1998). For each word in the dictionary, its synonyms are associated with the corresponding word vectors to obtain a synonym-word vector mapping. For the class label of each class, find out the words that are synonymous with the class label from the dictionary and obtain their corresponding word vector representations. These word vectors are then fused with the word vectors of the class labels of the original vectors, and the fused vectors are used as the new prototype vectors of the classes. When training on the samples, we process the data by applying the multi-head attention mechanism to make full use of its global perceptual capabilities to capture long-distance dependencies. To further enhance this capability, we introduce positional encoding in the model. Position encoding allows each attention “head” to dynamically adjust its attention level according to the position of the word in the sequence while paying attention to the local context of the current word, so as to pay attention to both the local and the overall situation, effectively grasp the structure and semantics of the entire input sequence, and thus achieve a more comprehensive and in-depth understanding of the input sequence.

The main contributions of the article are summarized as follows: (1) We utilize the global perception capability of the multi-head attention mechanism to capture long-distance dependencies, so that the model not only focuses locally, but also takes into account the whole situation, and is able to provide a deeper understanding of the input sentence sequence. (2) This article applies the few-shot classification technology to the field of social disputes for the first time. The speed and accuracy of classification provide favorable support for subsequent mediation, arbitration, trial, *etc*., greatly shortening the dispute handling time and improving the efficiency of dispute resolution. (3) We evaluate the model on four baseline datasets and one private dataset, the results show that our proposed model outperforms other baseline models.

## Related work

### Social disputes

Social disputes refer to the imbalance, conflict, confrontation, disorder and friction between individuals and groups (Chen, 2024). They cover the contradictions and disputes caused by various reasons in all aspects of social life, involving multiple fields such as economy, politics, culture and social life. Common types include:

**Civil disputes** social disputes between equal subjects with civil rights and obligations as their content, which are tractable. For example, disputes over property relations, such as contract disputes, property ownership disputes, property transfer disputes, *etc*.; disputes over personal relations, such as divorce disputes, personality rights disputes, identity disputes, *etc*.

**Administrative disputes** usually disputes between administrative counterparts and administrative subjects arising from administrative management activities. For example, citizens, legal persons or other organizations are dissatisfied with specific administrative actions such as administrative penalties, administrative licenses, and administrative enforcement made by administrative agencies, or believe that the administrative actions of administrative agencies infringe their legitimate rights and interests.

**Labor disputes** disputes between workers and employers arising in the labor process. Common ones include labor contract disputes, labor remuneration disputes, social insurance disputes, work-related injury compensation disputes, and labor contract termination disputes.

**Neighborhood disputes** generally occur between neighboring residents, and may involve neighboring rights issues, such as disputes over ventilation, lighting, drainage, and passage, or conflicts caused by trivial matters in life, noise interference, environmental sanitation, *etc*.

### Meta-learning

Few-shot text classification focuses on achieving high-precision classification when samples are scarce, and is a key research direction in the field of natural language processing. Few-shot learning tasks are usually set in the form of N-way K-shot, where N-way means that there are N different categories in the classification task, and K-shot means that each category has only K training samples. When faced with few-shot situations, traditional machine learning methods are often prone to overfitting due to the lack of sufficient data for learning, and it is difficult to accurately generalize to new samples. In order to break through this dilemma, researchers introduced the concept of meta-learning.

Meta-learning, also known as “learning to learn”, is based on the core idea of using the experience accumulated by the model on multiple tasks. This enables the model to quickly adapt to new tasks, especially in the case of few samples. The goal of meta-learning is not to directly optimize the performance of the model on a specific task, but to enable the model to master a general learning strategy so that when faced with new tasks, it can quickly adjust its own parameters or learning methods to achieve rapid learning. Few-shot learning is closely related to meta-learning. Meta-learning provides an effective solution for few-shot learning and is an important way to achieve few-shot learning. Through meta-learning, the model can be trained on multiple few-shot tasks, learn the commonalities and differences of these tasks, and then quickly adapt to new few-shot tasks using previously accumulated experience.

In the field of meta-learning, there are mainly three methods based on models, metrics, and optimization:

#### Model-based methods

The core of this approach is to achieve task adaptation by designing dynamic models with internal memory or fast parameter generation capabilities to directly update the hidden state using a small number of samples. Typical methods include memory-augmented neural networks (MANNs) (Santoro et al., 2016), MetaNet (Munkhdalai & Yu, 2017), and so on. MANNs are designed to achieve fast meta-knowledge migration and task adaptation in few-sample scenarios by dynamically reading/writing external memory to store task-related patterns. MetaNet learns inter-task shared knowledge in the meta-training phase by dynamically generating modularized network parameters with a fast weighting mechanism, which enables the model to quickly adapt to key parameters. Wang et al. (2023) improves cross-task generalization by incorporating structural causal modeling (SCM) to decouple causal and non-causal factors associated with tasks.

#### Metric-based methods

The core of this method is to learn a suitable distance or similarity function so that the model can make decisions directly based on the intrinsic relationship between input samples, without relying on a large amount of training data to learn complex discriminant boundaries. Such methods have important applications in few-sample text classification because they can effectively capture the similarity between samples and thus accurately classify them when samples are scarce. Among them, Sung et al. (2018) proposed a relational network, the core of which is to learn the relationship between samples. In few-shot text classification, it maps both the input text sample and the category prototype into a relational space. Snell, Swersky & Zemel (2017) proposed a prototypical network, which determines the category by calculating the distance between the sample and the prototype vector. Ma et al. (2022) by associating ProtoNet with Voronoi diagrams and optimizing the cluster structure, the accuracy and robustness of few-shot learning are improved, and the combined approach’s mathematical framework enhances overfitting resistance and supports geometric inference.

#### Optimization-based methods

The core of this approach is the design and optimization of model parameters to improve model adaptability and generalizability. Representative methods include model-agnostic meta-learning (MAML) (Finn, Abbeel & Levine, 2017) and Reptile (Nichol & Schulman, 2018). MAML learns generic initialization parameters by training on multiple tasks, which enables the model to converge quickly on new tasks and reduce the number of samples required for training. However, MAML optimizes the initial parameters using second-order gradients, which is computationally expensive. The Reptile algorithm simplifies this to a first-order approximation, utilizing task training weights with constant updates to optimize the initialization parameters, significantly improving training efficiency. Chijiwa et al. (2022) proposes the meta-ticket method, which dynamically searches for optimal sparse subnetworks for few-shot tasks from randomly initialized neural networks *via* a meta-learning framework, suppresses the overfitting risk of over-parameterized networks, and demonstrates better meta-generalization ability than MAML in large neural networks. Additionally, Kalais & Chatzis (2022) introduces an local winner-takes-all (LWTA)-based meta-learning method to generate sparse representations through random competition of neurons within a block, combining probabilistic modeling to suppress few-shot overfitting and enhance the model’s robustness to task uncertainty.

## Proposed method

In our research, we introduce a modeling framework that combines a meta-learning Siamese network as well as a multi-head attention mechanism to solve the paradoxical classification problem.

The meta-learning Siamese network, which consists of two sub-networks with shared weights, each of which is responsible for processing an input text. During the training process, the model learns how to map texts that represent similar contradictory properties to similar feature representations and texts that represent different contradictory properties to different feature representations. To achieve this, the meta-learning Siamese network uses a contrast loss function, which causes the model to distance samples of the same category closer together and push samples of different categories farther apart.

The multi-head attention mechanism further enhances the model’s representational power. The multi-head attention mechanism allows the model to focus on different parts of the text from a number of different viewpoints, thus capturing richer contextual information. This is particularly important for dealing with long texts and situations where long-distance dependencies need to be captured. By incorporating a multi-attention mechanism into the branch of the meta-learning Siamese network, we are able to ensure that the model not only learns local features in the text but also captures the overall semantic structure.

Therefore, the model combines the capabilities of the meta-learning Siamese network and multi-headed attention mechanism to efficiently deal with the complexity and diversity of the data in the text. The meta-learning Siamese network implements comparative learning to enhance the classification performance, and the multi-headed attention mechanism captures the long-distance dependency relationships, we have constructed a model that is both efficient and adaptable model. The streamlined architecture not only improves the training speed but also enhances the model’s adaptability to specific datasets, especially small datasets.

## Methods

### Problem setup

In a meta-learning classification task, we aim to find a function F based on the training task T and the corresponding training data D. F can output a function f, and f can be used for a new task. So the basic unit of meta-learning is the task, and meta-learning generally divides the data into a meta-training set ${y_{train}}$, and a meta-testing set ${y_{test}}$, where the training set and the testing set satisfy ${y_{train}} \cap {y_{test}} = \emptyset$. We aim to use data from meta-training with labels for model training, allowing the model to predict data that does not contain labels in the meta-test phase.

### Overview

**Figure 1:**
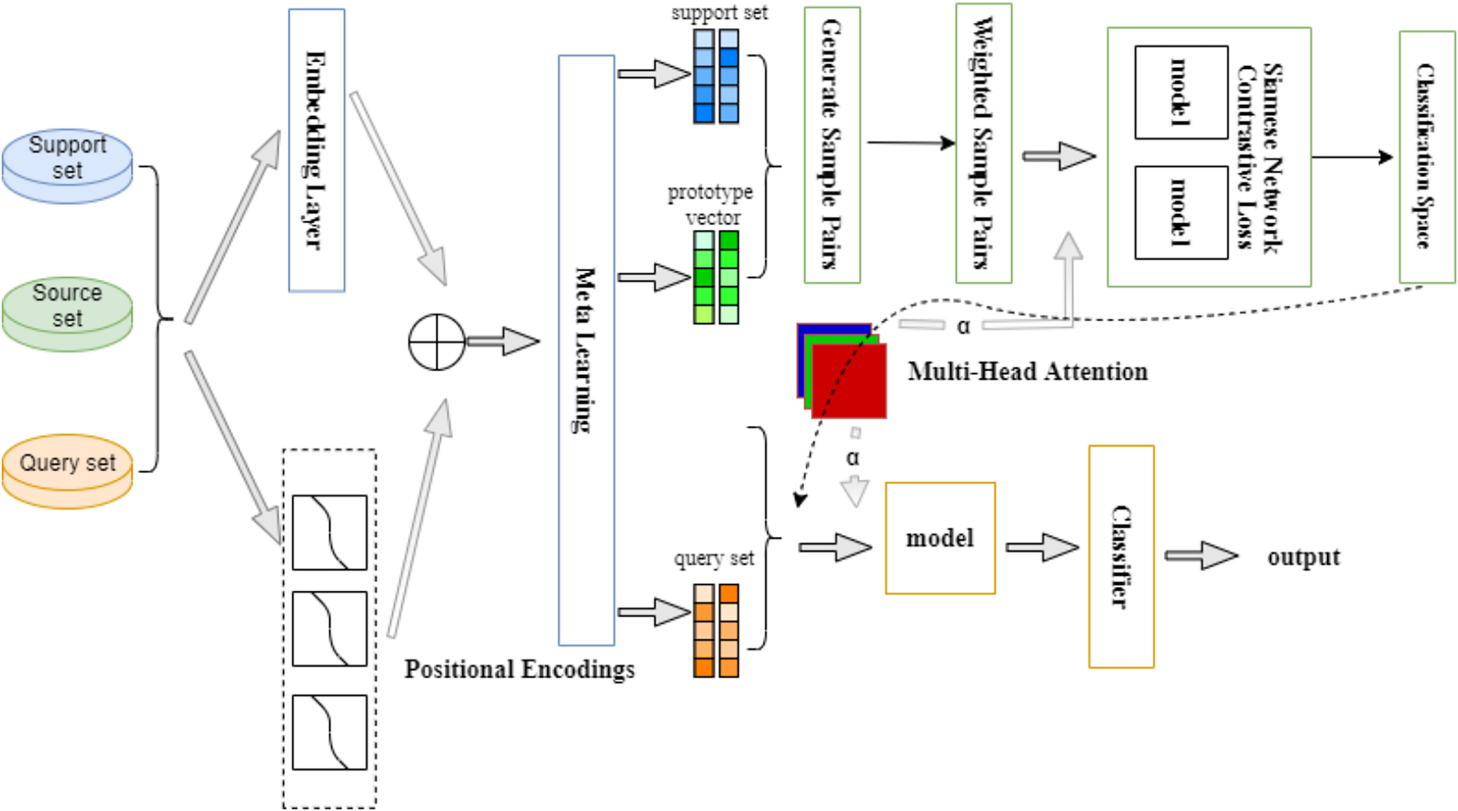
Multi-head attention Siamese meta-learning model architecture.

In our work, we adopt a method that combines meta learning Siamese network and multi-head attention mechanism. The meta learning Siamese network generates a low-dimensional space that is easier to classify. Through the multi-head attention mechanism, the model can learn the different associations of features in different attention heads, which allows the model to be more flexible in modeling based on the similarities and differences between different samples. Figure 1 shows an overview of the model proposed in this article.

We still use fastText as a pre-trained language model to generate d-dimensional word vectors. At the same time, to help the model better understand the relationship between words, we apply a position encoding module to encode the absolute and relative positions of words. Meta-tasks are then constructed from the training set, assigning higher probabilities to tasks that are more difficult to classify. After completing the construction, sample pairs are allowed to be generated between prototype vectors and prototype vectors, as well as between support sets and prototype vectors, and weights are generated for each sample. The core component Siamese network is responsible for mapping these samples and prototype vectors into a feature space that is more conducive to classification. We then apply the multi-head attention module to further enhance the model’s ability to focus on key information by classifying the query samples and prototype vectors into multiple subspaces, utilizing each subspace’s own weight matrix, and viewing the input data from different perspectives so that the model does not become overly reliant on a single type of data.

### Data preprocessing

The training, validation and test sets are first identified based on the specified dataset type. Then, the dataset is loaded from the JSON file and each row is converted into a dictionary containing labels and text content. Once the data is loaded, the vocabularies are constructed using pre-trained word vectors including special tokens such as $\lt$pad $\gt$ and $\lt$unk $\gt$, which denote filler words and unknown words, respectively. The vocabulary is constructed from all words that occur at least five times in the dataset. After initializing the vocabulary, the data is divided into training, validation, and test sets based on predefined class labels.

To enhance the robustness of the model, in the preprocessing stage, we constructed a dictionary of synonyms using WordNet and replaced some words in the dataset with synonyms. This step increases the diversity of the input data and helps the model to generalize better. In addition to this, this phase generates positional codes using the sine and cosine functions. These encoding will be added to the token embedding to let the model know the position of each word in the sentence. Next, the data is converted into a format suitable for training. This involves mapping each word in the text to its corresponding index in the vocabulary, ensuring that sequences are padded or truncated to a uniform length. Sequences consisting only of unknown or filled tokens are removed. For the training set, positional encoding is added to the token indexes to create a representation containing the identity and position of each token.

Finally, the data is organized into numpy arrays and returned with the vocabulary. This process converts the raw text into a structured numeric representation, making it ready to be fed into a machine learning model. The generated dataset includes text with positional coding, text length, labels, and other metadata needed to train and evaluate the model. In addition, class names were sorted to ensure consistency across script runs. The whole process ensured that the data was prepared in a way that took full advantage of word embedding and positional information but also mitigated the effects of a lack of vocabulary through synonyms.

### Position encoding

In natural language processing tasks, the order between words or symbols is very important. We often use both absolute and relative positional coding, in this article we use absolute positional coding, using sine and cosine functions to construct positional coding (Vaswani, 2017). This ensures that the model is able to handle sequences of different lengths and has the ability to capture long distance dependencies in the sequence, for each position pos and each dimension i, the position encoding PE(p, i) can be defined as follows:

$\eqalign {& PE(p,2i) = sin \left({{pos} \over {{{10\hbox{,}000}^{2i/d}}}}\right) \\ & PE(p,2i + 1) = cos \left({{pos} \over {{{10\hbox{,}000}^{2i/d}}}}\right)}$

where d is the dimension of the position encoded vector and i is the position index in the position encoded vector.

### Multi-head attention

**Figure 2:**
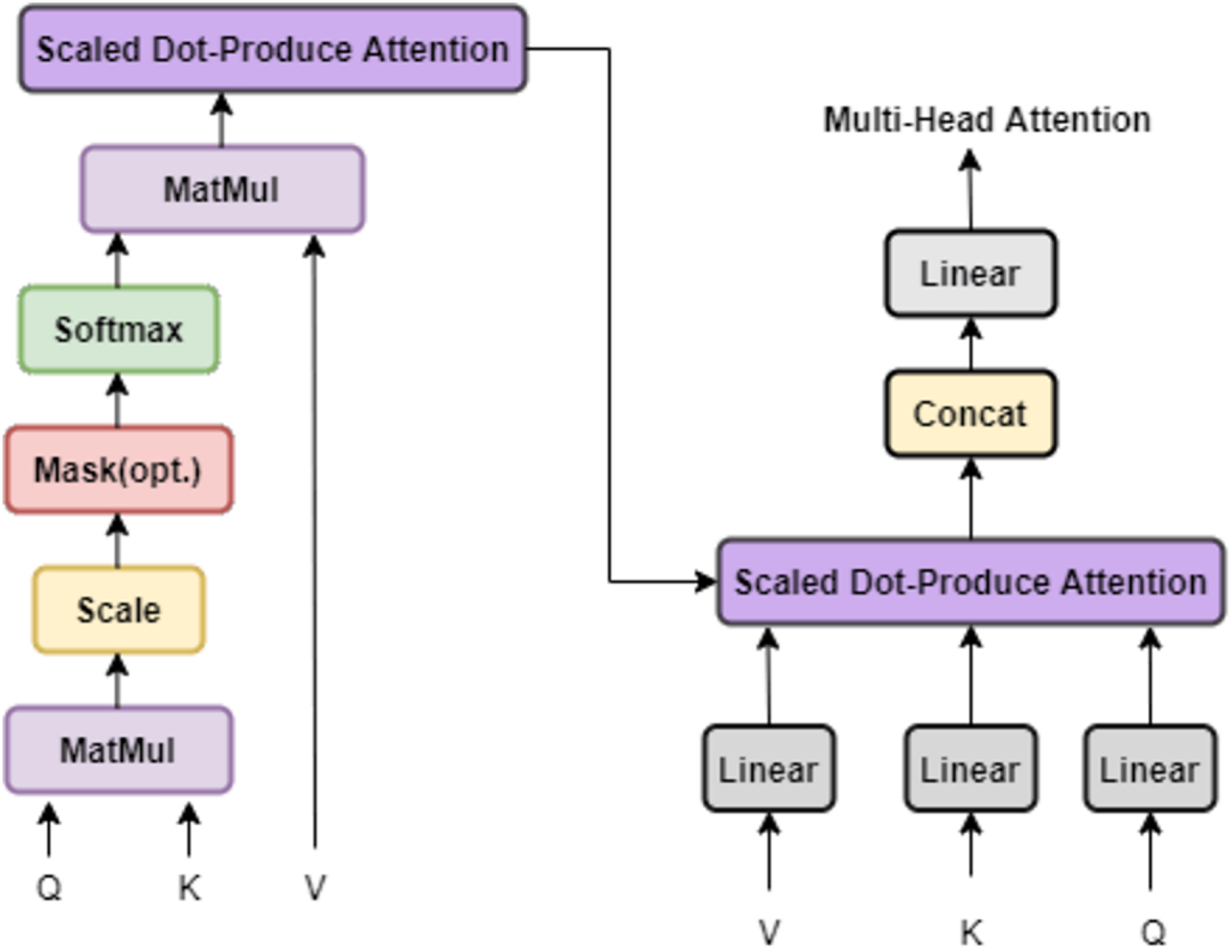
Multi-head attention.

The core of the multi-head attention mechanism is to enhance the expressive power of the model by running multiple self-attention layers in parallel and synthesizing their results, which can simultaneously capture the information of the input sequence in different subspace. Specifically, multi-head attention processes the input sequence through multiple parallel attention layers, with each “head” having its weight matrix, so that the input data can be viewed from different perspectives. After each head completes an attention operation, the results will be spliced through a linear layer again to obtain the final attention output. The specific structure is shown in Fig. 2.

Below we describe the tasks accomplished by the multiple attention mechanism in this article. For the input data, each word is first mapped to a d-dimensional vector using the pre-trained language model fastText (Joulin et al., 2016). Then, we obtain the sequence information by adding positional encoding and perform meta-task sampling by performing meta-task construction on the word vectors to get the query set ${X_{i}} = [{x_{i1}},{x_{i2}}, \ldots ,{x_{in}}]$. Not all words in a sentence are equally important to the meaning representation of the sentence, so we use the attention mechanism to focus on the more meaningful words.

$${ \alpha _{it}} =  {{exp(x_{it}^T{a_{w}})} \over {\sum\nolimits_{t = 1}^{{N_{i}}} e xp(x_{it}^T{a_{w}})}} \\ {s_{i}} =  \sum\limits_{t = 1}^{{N_{i}}} {{\alpha _{it}}} {x_{it}}. $$

First, we quantify the importance of each word by computing the similarity between ${x_{it}}$ and the word-level attention vector ${a_{w}}$ and normalizing it using the attention weights $\alpha$ it. Next, we construct the sentence representation ${s_{i}}$ based on these weights, which is a weighted sum of the context word representations. In this process, the attention vector ${a_{w}}$ actually acts as a query to help us determine which words are valuable for classification.

However, relying only on a single attention vector to select features has its limitations. In order to select features required for different prediction classes in a more targeted manner, we introduce a multiple attention mechanism. This mechanism allows the model to simultaneously attend to information from different representation subspace, which in turn enhances the effectiveness of the attention mechanism. In short, we use multiple attention vectors to form respective focused attention word representations, which enriches feature selection and classification accuracy.

### Meta-learning siamese network

The meta-learning Siamese network aims to reduce the model’s reliance on the single representation of the initial class label and alleviate over-reliance on the accuracy of external knowledge of the class label. Therefore, in this method, we no longer rely solely on the original class label representation, but search for a list of synonyms for each class label. After obtaining the word vector representations of these synonyms, we continue to expand the prototype vector representation by considering the weights of the synonyms.

Firstly, the model maps the words in the text to d-dimensional vectors using the pre-trained language model fastText (Joulin et al., 2016). Then we use WordNet (Fellbaum & Miller, 1998) to obtain a dictionary of synonyms, assuming that for the word ${w_{i}}$, the list of synonyms is ${w_{i}} = \{ {w_{i1}},{w_{i2}}, \ldots ,{w_{in}}\}$, and the corresponding word vector is ${c_{i}}$, from which we obtain the synonym-word vector mapping ${w_{ij}} = {c_{ij}}$. Next, we find the thesaurus of synonyms for each class label, obtain the word vector representation, and extend the prototype vector representation according to the weights of the synonyms. So for each category ${c_{i}}$, the new prototype vector after fusion is:

$\matrix{f_{0}}({c_{i}}) = {1 \over N}\sum\limits_{i = 1}^N {w_{ij}^*} {c_{ij}}$

where N is the number of synonyms and $w_{ij}^*$ is the weight of synonym ${w_{ij}}$. The weight $w_{ij}^*$ of each synonym ${w_{ij}}$ is determined through a multi-step process that combines semantic similarity and frequency information.

First, we calculate the semantic similarity between each synonym ${w_{ij}}$ and the original class label word ${w_{i}}$. We use the path-based similarity algorithm provided by WordNet. The semantic similarity score $si{m_{path}}({w_{ij}},{w_{i}})$ is computed as:

$si{m_{path}}({w_{ij}},{w_{i}}) = {{2 \times depth(LCS({w_{ij}},{w_{i}}))} \over {pathLength({w_{ij}},{w_{i}}) + 2 \times depth(LCS({w_{ij}},{w_{i}}))}}.$

Here, $depth(LCS({w_{ij}},{w_{i}}))$ denotes the depth of the lowest common subsumer (LCS) of ${w_{ij}}$ and ${w_{i}}$ in the WordNet hierarchy, and $pathLength({w_{ij}},{w_{i}})$ represents the shortest path length between ${w_{ij}}$ and ${w_{i}}$ in WordNet. A higher semantic similarity score indicates a closer semantic relationship between the synonym and the original class label word.

In addition to semantic similarity, we also take into account the frequency of each synonym ${w_{ij}}$ in the dataset, denoted as $freq({w_{ij}})$. The frequency reflects how often the synonym appears in the context of the class, which can be an important indicator of its relevance. We then combine the semantic similarity score and the frequency information through a weighted sum to obtain a comprehensive score for each synonym. Based on experimental results, we assign a weight of 0.6 to the semantic similarity score and 0.4 to the frequency score. The comprehensive score $score({w_{ij}})$ is calculated as:

$score({w_{ij}}) = 0.6 \times si{m_{path}}({w_{ij}},{w_{i}}) + 0.4 \times freq({w_{ij}}).$

Finally, to ensure that the weights of all synonyms sum up to 1, we perform a normalization process. The weight $w_{ij}^*$ of each synonym ${w_{ij}}$ is obtained by dividing its comprehensive score by the sum of the comprehensive scores of all synonyms:

$w_{ij}^* = {{score({w_{ij}})} \over {\sum\nolimits_{j = 1}^N s core({w_{ij}})}}.$

Similarly, the initialized prototype vectors for sample ${s_{i}}$ can be obtained in an analogous manner. By integrating synonym information and carefully calculating the weights of synonyms, Meta-SN is able to construct more comprehensive and accurate prototype vectors, which significantly enhances its performance in few-shot text classification tasks.

Next, to better construct meta-tasks, this article optimizes the meta-learning task in few-shot text classification, which focuses on the sampling probability of meta-tasks where the distances between prototype vectors are closer and where the samples are farther away from the corresponding prototype vectors. Therefore, we first define a probability $p_{i,j}^c$, it calculates the correlation between ${c_{i}}$, ${c_{j}}$ for class selection during task sampling, the closer the distance, the higher the correlation and the higher the probability of being selected, where dis denotes the distance vector and|*C*| is the total number of classes. Then, for a known class ${c_{i}}$, a probability score $p_{i,j}^s$ is set. The distance between the sample and the corresponding prototype vector is calculated; the longer the distance, the higher the sampling probability, so as to assign a higher sampling probability to samples that are difficult to classify and improve the generalization ability of the model.

$\eqalign{p_{i,j}^c =\; & {{{e^{ - dis({f_{0}}({c_{i}}),{f_{0}}({c_{j}}))}}} \over {\sum\nolimits_{k = 1}^{|C|} {{e^{ - dis({f_{0}}({c_{i}}),{f_{0}}({c_{k}}))}}} }}\\ p_{i,j}^s = \;& {{{e^{dis({f_{0}}({c_{i}}),{f_{0}}({s_{j}}))}}} \over {\sum\nolimits_{k = 1}^{|C|} {{e^{dis({f_{0}}({c_{i}}),{f_{0}}({s_{k}}))}}} }}.}$

From this, we can construct the meta-task. First, an initialization class is randomly selected, and then other classes are gradually added based on the correlation probabilities $p_{i,j}^c$ between classes, with a preference for classes that are closer to existing classes. For each selected class, the sampling of samples in the support set and query set is determined by $p_{i,j}^s$, with preference given to samples that are far from the prototype vectors to ensure the difficulty and diversity of the meta-task.

After sampling the meta-tasks, the generation of sample pairs starts. Firstly, the prototype sample pair is generated, which is $\lt{p_{i}},{p_{j}} \gt$, and then the support set ${s_{i}}$ is generated with the prototype vector ${p_{j}}$ sample pairs, which is denoted as $\lt{s_{i}},{p_{j}} \gt$, and if the two samples in a sample pair belong to the same class, then the label for this pair is 1; otherwise, it is 0. The weights are generated for each sample, which is based on the inverse of the average distance between the samples and the query set. The weights of the pairs of samples in the support set are normalized using the softmax function so that samples that are closer to the query set have higher weights, the prototype vectors and samples are further refined by the Siamese network.

${\omega _{\lt{s_{i}},{\emptyset _{j}} > }} = softmax \left[ - {1 \over L}\sum\limits_{i = 1}^L d is({f_\theta }({s_{i}}),{f_\theta }({q_{l}}))\right].$

Optimizing the representation of samples and prototype vectors by Siamese network, the Siamese network contains two structurally identical branches dealing with samples and prototype vectors respectively, mapping them by means of shared weights into a low-dimensional space where samples of the same kind are closer together and the distance between samples of different classes is greater. Finally, the Siamese network is optimized using the contrastive loss function.

${L_{c}}(\theta ) = \sum\limits_{i = 1}^n {{\omega _{\lt{x_{il}},{x_{i}}r > }}} [{y_{i}}dis({f_\theta }({x_{il}}),{f_\theta }({x_{ir}})) + (1 - {y_{i}})max(0,\delta - dis({f_\theta }({x_{il}}),{f_\theta }({x_{ir}})))]$

## Experiments

### Datasets

In our experiments, we used four benchmark datasets HuffPost, Amazon, 20 Newsgroups, Reuters and a private dataset of conflicts and disputes.

HuffPost headlines consist of news headlines published on HuffPost between 2012 and 2018 (Misra, 2018). These headlines are divided into 41 classes. They are shorter and less grammatical than formal sentences.

20 Newsgroups consist of informal discourse in news discussion forums (Lang, 1995). The document consists of 20 topics.

Amazon product data contains product reviews for 24 product classes, with 142.8 million reviews spanning the period 1996–2014 (He & Mcauley, 2016). Our task is to identify the product classes of the reviews. Due to the large size of the original dataset, we extract 1,000 reviews from each class as a subset.

Reuters is collected from Reuters articles in 1987. We use the standard ApteMode version of the dataset. Following (Bao et al., 2019), we evaluate 31 classes and eliminate articles with multiple labels. Each class comprises a minimum of twenty articles.

A private dataset constructed in this study aims to provide targeted data support for text classification research related to social disputes. The data mainly comes from case information disclosed by the judicial network and relevant reports released by various media. From these data sources, we carefully extracted key information related to social disputes, which covers multiple dimensions such as dispute titles, dispute types, and dispute details.

In terms of types of disputes, we summarized a total of 19 different types of disputes after careful sorting and integration. For example, medical disputes: The family of a patient sued a hospital alleging a misdiagnosis resulting in delayed treatment (*e.g*., ‘appendicitis misdiagnosed as gastroenteritis’). Labor dispute: Factory workers collectively claimed compensation for occupational diseases caused by long-term exposure to hazardous substances. Consumer dispute: Users of an online shopping platform reported the sale of counterfeit products with falsified quality certificates. Property dispute: Dispute over the right to use shared building access by neighboring owners in a residential neighborhood.

In the sorting process, we standardized similar data types, for example, classifying both “road traffic accident disputes” and “traffic accident disputes” as “traffic accident disputes” to ensure data consistency and accuracy. These 19 types of disputes basically cover common social dispute scenarios, providing a rich data foundation for the classification performance of the research model for different types of disputes.

In terms of the dataset scale, we included a total of 10,316 data records. These data records detail the specific circumstances of various social disputes. The title of the dispute briefly summarizes the core issues of the dispute, and the details of the dispute contain rich information such as the background, process, and personnel and institutions involved in the disputes, providing strong support for in-depth analysis of the content of the dispute.

### Baselines

To evaluate the performance of our model, we compare it with other meta-learning models: MAML (Finn, Abbeel & Levine, 2017) learns easily adaptable model parameters by gradient descent. The protocol network (Snell, Swersky & Zemel, 2017) learns a metric space in which classification is performed by computing the Euclidean distance to the prototype representation of each class. Induction networks (Geng et al., 2019) leverage the dynamic routing algorithm in meta-learning to learn class-level representations in the task of few-shot text classification, and enables the model to tackle the few-shot classification challenge more effectively by generalizing and generalising class representations in the support set. Gao et al. (2019) proposes a hybrid attention prototype network (HATT) approach for the problem of classifying few-shot relations in noisy environments, which strengthens the model’s expressiveness and robustness in noisy text by designing instance-level and feature-level attention mechanisms. The Distributional Signatures Few-Shot Learning (DS-FSL) (Bao et al., 2019) method uses the importance of lexical distributional features to improve the few-shot classification by mapping the distributional features to attention scores, which in turn guides the model to quickly adapt to new classification tasks. The MetaLearning Adversarial Domain Adaptation (MLADA) (Han et al., 2021) introduces the idea of GAN into few-shot learning by adding meta-knowledge generator and discriminator structures to recognize important lexical features and generate high-quality sentence embedding in new classes to improve few-shot classification. Hong & Jang (2022) proposed LEA, which effectively facilitates the application of large pre-trained language models to few-shot learning by constructing meta-level attention features across tasks *via* a meta-learning framework and inferring the most relevant attention aspects when encountering a new task. The meta-learning Siamese network (Meta-SN) (Han et al., 2023) improves the generalization ability of the model under limited sample conditions by constructing prototype vectors using external knowledge of class labels and focusing on difficult-to-classify samples in combination with a well-designed sampling strategy.

### Implementation details

Based on previous work, we use the pre-trained model fastText (Joulin et al., 2016) for word embedding. The model was updated using the Adam (Kingma & Ba, 2014) optimizer, and the initial learning rate was set to 0.002. The cross-entropy loss function was used to calculate the loss in model training with an initial value of 0.00002. In the Siamese network, we use a 2D filter of size ([2, 3, 4], embedding dim) with a dimension of 64 for the fully connected layer. Some of the results in the article come from the original articles of the models used. In this article, we follow the same train/val/test split as MLADA. In the meta-training phase, 100 training episodes are performed per epoch, while stopping early if the accuracy on the validation set does not increase after 20 epochs. We evaluate the model performance based on 1,000 meta-tasks in meta-test and give the average accuracy over five runs. All experiments are performed on a NVIDIA v100 GPU.

In order to optimize the performance of the model, we have carefully tuned several key hyperparameters. Defining the hyperparameters to be tuned and their value ranges is the first step in hyperparameter selection. We focus on the following hyperparameters and their settings.

#### Number of attention heads

The number of attention heads is a key factor affecting the model’s ability to parallelize processing in the attention mechanism. Different numbers of attention heads allow the model to capture text features from different subspaces, thus improving the model’s understanding of text semantics. We set the number of attention heads to one, two, four, eight, and 16 to investigate the effect of different numbers of attention heads on the model performance.

#### Learning rate

The learning rate controls the step size of parameter update during the training process. A suitable learning rate can enable the model to converge to the optimal solution quickly during the training process, preventing it from skipping the optimal solution due to a large step size. We set the initial learning rate to 0.002, and then use the ReduceLROnPlateau strategy to dynamically adjust the learning rate. This strategy, based on the model’s performance, automatically reduces the learning rate when the accuracy on the validation set does not improve significantly within a certain number of rounds, in order to help the model jump out of the local optimum and continue to converge to the global optimum.

In order to find the optimal hyperparameter combinations from the above hyperparameter search space, we employ a grid search method. Grid search is a systematic and comprehensive hyperparameter search strategy that traverses all possible combinations in the hyperparameter search space. ReduceLROnPlateau dynamically adjusts the learning rate based on the performance of the validation set during the training process. For each value of the number of attention heads, we conduct a complete training process to allow ReduceLROnPlateau to play its role. Ultimately, we obtain the model’s performance under each combination.

### Experimental results

The results of the experiments on the benchmark dataset are shown in Table 1. The results on the private dispute dataset are shown in Table 2. Our model outperforms previous models on all datasets, except in 1-shot Amazon. The average accuracy is 73.4% in 1-shot classification and 87.3% in 5-shot classification, which is 9.2% better than the state-of-the-art Meta-SN model. It proves the effectiveness of our multi-attention mechanism application. Specifically, there is a 8.0% and 8.3% improvement in 1-shot and 5-shot on HuffPost, and a 3.9% improvement in 5-shot on Amazon, and a 9.2% and 3.0% improvement in 1-shot and 5-shot on 20 News, and a 7.7% and 1.7% improvement in 1-shot and 5-shot on Reuters.

**Table 1:** Results of 5-way 1-shot and 5-way 5-shot classification on four benchmark data sets.

Methods	HuffPost		Amazon		20 News		Reuters	
	1-shot	5-shot	1-shot	5-shot	1-shot	5-shot	1-shot	5-shot
MAML	35.9	49.3	39.6	47.1	33.8	43.7	54.6	52.9
ProtoNet	35.7	41.3	37.6	52.1	37.8	45.3	59.6	66.9
Induct	38.7	49.1	34.9	41.3	28.7	33.3	59.4	67.9
HATT	41.1	56.3	49.1	66.0	44.2	55.0	43.2	56.2
DS-FSL	43.0	63.5	62.6	81.1	52.1	68.3	81.8	96.0
MLADA	45.0	64.9	68.4	86.0	59.6	77.8	82.3	96.7
LEA	46.2	65.8	66.5	83.5	54.1	60.2	69.0	89.0
Meta-SN	54.7	68.5	**70.2**	87.7	60.7	78.9	84.0	97.1
MASM	**62.7**	**76.8**	69.1	**91.6**	**69.9**	**81.9**	**91.7**	**98.8**

**Note:**

The best results are shown in bold.

**Table 2:** The results on the private dispute dataset.

Models	Accuracy		Recall		F1-score	
	1-shot	5-shot	1-shot	5-shot	1-shot	5-shot
Meta-SN	45.8	56.3	45.7	56.3	44.9	56.1
MASM	**47.2**	**60.1**	**47.2**	**60.0**	**47.1**	**59.4**

**Note:**

The best results are shown in bold.

In addition to this, on the private dispute data, our MASM model demonstrated strong performance. It achieved an accuracy of 47.2% in the 1-shot scenario and 60.1% in the 5-shot scenario, outperforming the Meta-SN model which had 45.8% and 56.3% respectively. Similar trends were observed in recall and F1-score.

These results indicate that our MASM model can be well-applied to the conflict dispute domain. Its superiority across multiple key metrics in both 1-shot and 5-shot scenarios highlights its effectiveness in handling few-shot classification tasks related to social disputes.

**Figure 3:**
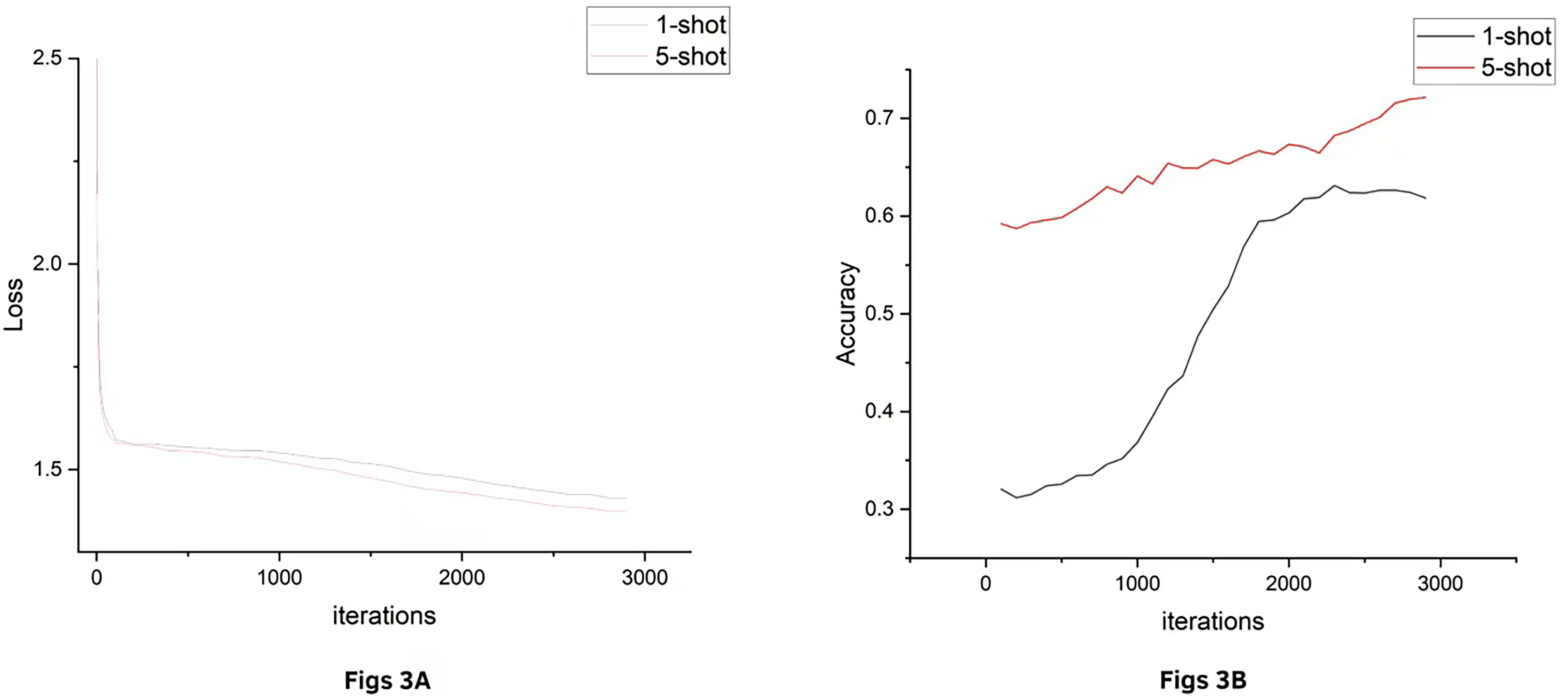
HuffPost dataset results.

In order to examine the overall performance of the model on the classification task as well as to assess the accuracy of the model’s predictions, we plotted images of accuracy and loss for the huffpost dataset, Fig. 3A shows the change in loss during training and Fig. 3B shows the increase in accuracy with training rounds on the validation set. The horizontal axis represents the number of training rounds, the vertical axis in Loss represents the loss value, and the vertical axis in Accuracy represents the classification accuracy. It can be seen that the loss values both decrease gradually with training until they plateau, accuracy quickly reaches high accuracy in the 1-shot condition and continues to grow steadily in the 5-shot condition. For other datasets, there will be similar results, so they are omitted in this article.

### Ablation studies

We conducted ablation studies to validate the effectiveness of the proposed multi-head attention and synonym substitution. The validation was performed on the Huffpost dataset.

Firstly, we investigated how synonym substitution would affect our model results as shown in Table 3. The results demonstrate that the use of synonym substitution enhances the model’s understanding of category semantics, enabling the model to make more accurate classification decisions when faced with new samples. This experiment verifies the effectiveness of synonym substitution for model enhancement.

**Table 3:** Results of ablation experiments.

Models	Accuracy	
	1-shot	5-shot
- No synonym replacement	60.3	73.9
Attention head count		
- 1	56.6	69.2
- 2	61.5	75.4
- 4 (MASM)	**62.7**	**76.8**
- 8	62.1	75.7
- 16	61.4	74.9

**Note:**

The best results are shown in bold.

**Figure 4:**
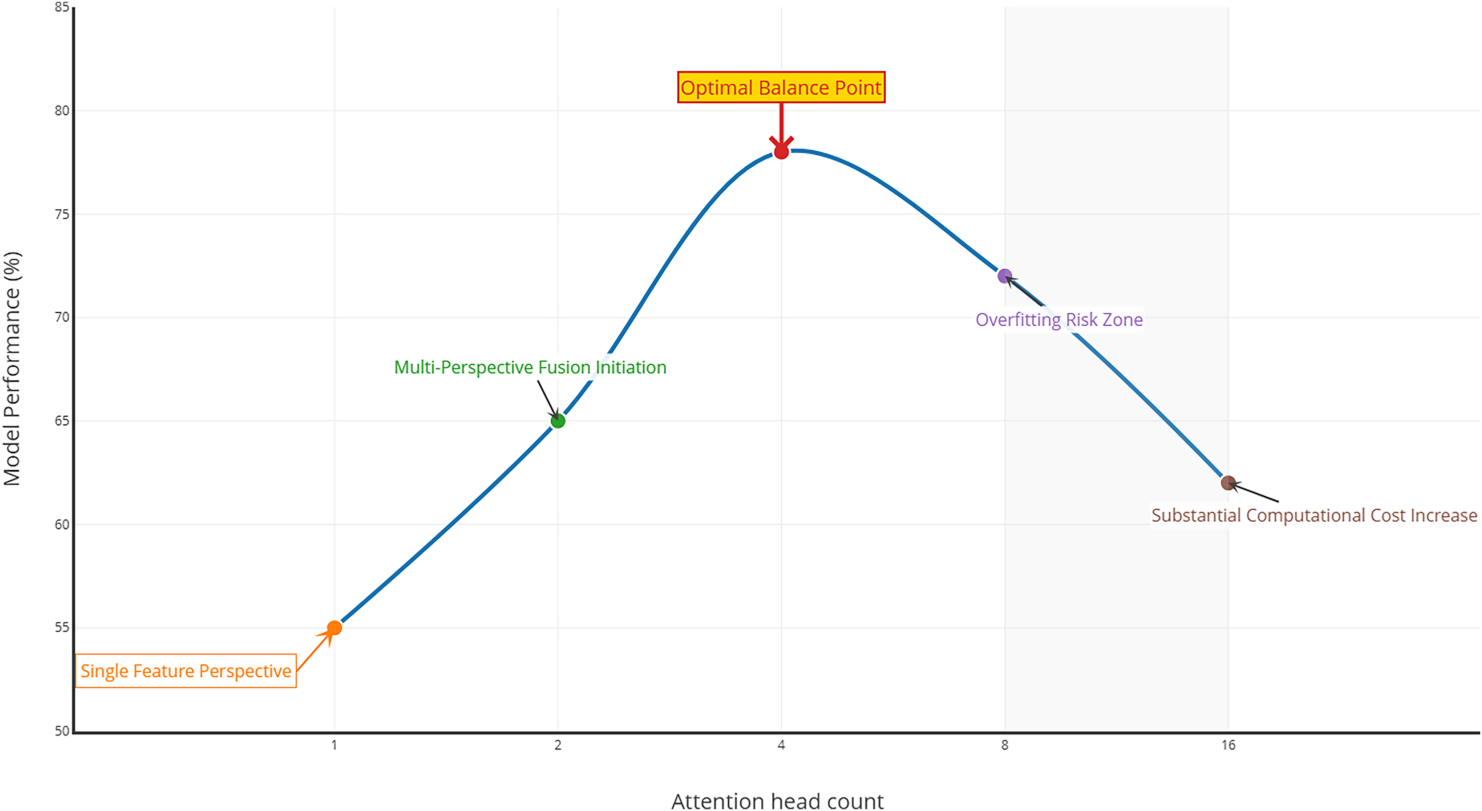
The effect of the number of attentional heads on model performance.

In addition, we explored the effect of different numbers of attention heads on the model performance, as shown in Fig. 4. When the number of attention heads is one, the model may only be able to focus on a single feature perspective of the text, and its performance is relatively low. In contrast, when the number is increased to two and four, the model can process feature representations from multiple different subspaces in parallel, leading to a more comprehensive understanding of the text and a significant performance improvement.

However, when the number of attention heads is further increased to eight and 16, the model performance starts to decline. This may be because too many attention heads significantly increase the model complexity, raise the number of parameters to be learned, and make the model prone to overfitting with limited training samples, resulting in poorer generalization ability. Moreover, too many heads also increase the computation amount and training time, reducing the training efficiency.

By considering the performance, computational resources, and training efficiency, we choose four attention heads. With these four heads, the model achieves optimal performance on both indexes and strikes a better balance between performance enhancement and resource consumption, enabling it to perform best in practical applications.

### Visualization

**Figure 5:**
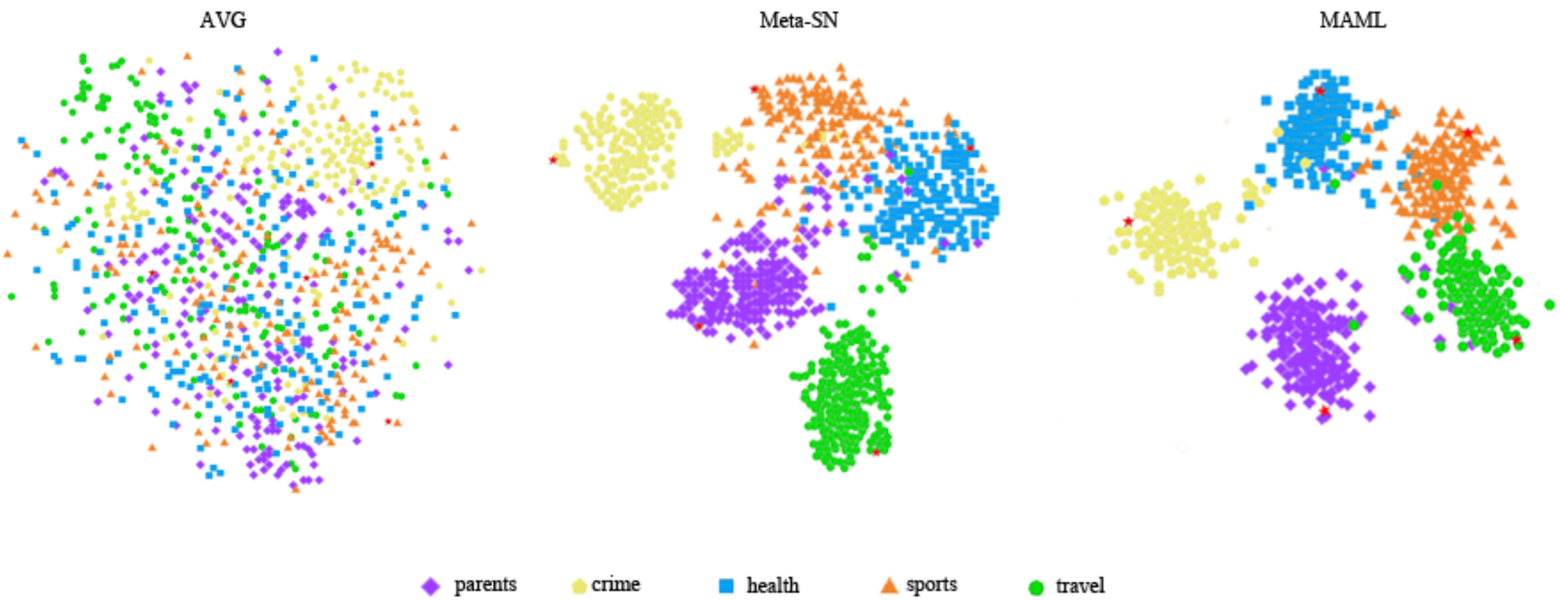
The t-SNE visualization comparison of sentence embeddings in metatesting on the HuffPost dataset.

Next, we used visualisation techniques to highlight the efficacy of various embedding methods in capturing semantic nuances of invisible class. To this end, we used the t-SNE (Laurens & Hinton, 2008) dimensionality reduction technique to visualize the sentence embeddings of the query set generated by different methods on the HuffPost dataset, as shown in Fig. 5.

AVG is used to generate sentence embedding by directly averaging the word embedding contained in the sentence. It is clear in the figure that the sentence embedding are entangled among the classes. Meta-SN generates better sentence embedding compared to AVG, but some classes are still entangled. Finally, MASM generates embedding that are more clearly separated. This demonstrates the superiority of our method in generating high-quality embedding.

## Limitations

Positional encoding was introduced to allow the model to better understand the positional information of each element in the input sequence. While positional encoding itself does not directly increase the number of parameters in the model, it requires an additional computational step to generate the corresponding encoded value for each position in the sequence. Increasing the computational burden on the model. In addition to this, the multi-head attention mechanism allows the model to attend to different parts of the input from multiple perspectives at the same time, which means that the model needs to maintain multiple parallel attention heads. Each attention head needs to independently compute the attention weights and then perform a weighted summation of the inputs. This architectural design allows the model to take more information into account when making sense of the input sequence, but it also correspondingly increases the overall complexity of the model.

## Conclusions

In this article, we propose a new multi-head attention Siamese meta-learning network for few-shot classification, which extends the representation of prototype vectors by synonym substitution, enhancing the model’s understanding of text semantics and classification performance. In addition, we also propose a multi-head attention mechanism that has allowed focusing on long-distance relational dependencies in the text rather than only relying on local knowledge and overreliance on a single type of feature representation; this reduces the risk of overfitting, and multi-head attention improves the generalization ability of the model by learning features from different subspaces. The proposed model is evaluated on four standard datasets outperforms other models on all datasets and achieves good performance on the dispute dataset, demonstrating the applicability of our model in the field of social disputes. However, the current work focuses on text. In the future, combining multimodal information such as images and videos could improve the model’s comprehension of complex scenes. Additionally, considering real-time demands in practical applications, the computational efficiency of the model should be optimized, and techniques like model compression and distillation can enable its deployment on mobile devices or edge computing environments.

## Data

Third party data used in this article includes HuffPost headlines (https://www.kaggle.com/datasets/rmisra/news-category-dataset), 20 Newsgroups (http://qwone.com/~jason/20Newsgroups/), Amazon reviews (https://cseweb.ucsd.edu/~jmcauley/datasets.html#amazon_reviews), and Reuters-21578 (https://kdd.ics.uci.edu/databases/reuters21578/reuters21578.html).

## Supplemental Information

10.7717/peerj-cs.2910/supp-1 Supplemental Information 1 Core code for this experiment.

## Additional Information and Declarations

### Data Availability

The following information was supplied regarding data availability:

The data is available at Zenodo: Wang. (2024). few-shot dataset [Data set]. Zenodo. https://doi.org/10.5281/zenodo.13218559.
